# How to place the duality of specific MMP-9 inhibition for treatment of inflammatory bowel diseases into clinical opportunities?

**DOI:** 10.3389/fimmu.2022.983964

**Published:** 2022-09-09

**Authors:** Ghislain Opdenakker, Séverine Vermeire, Ahmed Abu El-Asrar

**Affiliations:** ^1^ Rega Institute for Medical Research, Department of Microbiology, Immunology and Transplantation, KU Leuven, Leuven, Belgium; ^2^ University Hospitals Leuven, KU Leuven, Leuven, Belgium; ^3^ Department of Ophthalmology, King Saud University, Riyadh, Saudi Arabia

**Keywords:** matrix metalloprotease, tissue inhibitors of metalloproteases, ulcerative colitis, Crohn’s disease, diabetic retinopathy

## Abstract

Crohn’s disease (CD) and ulcerative colitis (UC) are inflammatory bowel diseases (IBD) with the involvement of immune cells and molecules, including cytokines, chemokines and proteases. A previous extensive review about the molecular biology of matrix metalloproteases (MMPs) and tissue inhibitors of metalloproteases (TIMPs), related to intestinal barrier destruction and restoration functions in IBD, is here complemented with the literature from the last five years. We also compare IBD as a prototypic mucosal inflammation of an epithelial barrier against microorganisms with inflammatory retinopathy as a disease with a barrier dysfunction at the level of blood vessels. Multiple reasons are at the basis of halting clinical trials with monoclonal antibodies against MMP-9 for IBD treatment. These include (i) the absence of a causative role of MMP-9 in the pathology in animal models of IBD, (ii) the fact that endotoxins, crossing the intestinal barrier, induce massive local release of both neutrophil collagenase (MMP-8) and gelatinase B (MMP-9), (iii) insufficient recognition that MMPs modify the activities of cytokines, chemokines and their receptors, (iv) ignorance that MMPs exist as mixtures of proteoforms with different posttranslational modifications and with different specific activities and (v) the fact that MMPs and TIMPs act in an interactive network, possibly having also beneficial effects on IBD evolution. Nevertheless, inhibition of MMPs may be a useful therapeutic approach during specific IBD disease phases or in specific sub-phenotypes. This temporary “window of opportunity” for MMP-9 inhibition may be complemented by a locoregional one, provided that the pharmacological agents are targeted in time to affected tissues, as is achieved in ophthalmological inflammation. Thus, in order to discover spatial and temporal windows of opportunity for MMP inhibition as treatment of IBD, more preclinical work including well controlled animal studies will be further needed. In this respect, MMP-9/NGAL complex analysis in various body compartments is helpful for better stratification of IBD patients who may benefit from anti-MMP-9.

## Introduction

Three essentials of scientific research and progress are confirmation, comparison and complementation. These three essentials have been contested in studies of MMP-9 inhibition for IBD treatment. In such situations, it is not an option to give up basic or clinical research, but instead it should create opportunities for more and better studies and to reconsider original ideas, maybe in new contexts. A critical review about the biology, biochemistry and pathology of all MMPs and TIMPs in IBD was published in 2016 (1). This review contained extensive information and discussion about the benefits and limitations of animal models for IBD research, and it provided updates of diagnostic procedures and mechanistic insights into present-day therapies, including clinical trials with antibodies against MMP-9. We here review the recent literature related to MMPs in IBD since 2016. To renew and enhance interest, we here also compare and complement IBD studies with insights from analyses of MMPs and TIMPs in diabetic retinopathy (DR)). It is thereby our aim to stimulate further research with new technologies in order to understand the detrimental and beneficial roles of MMPs. This approach may help to define windows of opportunity for MMP-9 inhibition as treatment for IBD.

## What were the evidences to inhibit MMP-9 specifically in IBD?

A series of studies was at the basis of commercial investments to place MMPs and in particular MMP-9 as priority targets in the fight against IBD. With the use of association studies, linking genetic polymorphisms in MMP genes with IBD susceptibility, one overview (2) and one primary study (3) linked the locus rs1569723 to MMP-9. However, an in-depth analysis of all MMP genes (1, see Table 2 therein) and contrasting results of association studies in various patient cohorts, as well as contested studies about TIMP gene association studies with IBD, made us conclude that MMP and TIMP loci are clearly not associated with IBD, weakening the possibility that the studied MMPs may become simple therapeutic targets (1). Similar inconclusive findings were reviewed for the four human TIMP genes (1).

In the absence of genetic association, the next level is the study of altered mRNA expression of MMPs or TIMPs in association with IBD. Detailed analysis of mRNA expression with various methods yielded no conclusive data. The methods included qRT-PCR, gene arrays with various densities and *in situ* hybridization on intestinal cell lines and various primary intestinal cell cultures and on intestinal biopsy materials of patients with UC or CD and controls. Also these data were compiled until 2016 in a critical review (1) and are till today not revoked.

In addition to mRNAs, MMP and TIMP proteins have been extensively studied in IBD patients and in various animal models of acute and chronic colitis with inclusion of post-colitic fibrosis (1). One step too far into MMP-9 biology was the (wrong) thinking that if the protein levels are increased in IBD and important substrates of MMP-9 are cleaved *in vitro* and *in situ*, it has a primary causative role in IBD and its inhibition may restore the broken intestinal barrier. However, without contesting the facts that MMP-9 cleaves substrates at the intestinal barrier (denatured collagens, claudins, occludins, precursor defensins, actins, cadherins, the cytokines TNF and VEGF and the chemokine ligand CXCL-8/IL-8 and cellular receptors) (1, see Figure 3 therein), and may contribute mechanistically to tissue damage, this may be rather effect than cause. Stopping MMP-9 activity in such case, e.g. by diminishing its production or activation or by inhibition of its activity, will lead to less barrier breakdown, but also abolish its potential beneficial functions.

MMP inhibitors of various kinds, from small molecule inhibitory drugs to highly specific monoclonal antibodies (4, 5), have been developed, particularly for oncology research. For cancer treatment, these drugs failed so far and many explanations have been formulated for this, including lack of specificity of small molecular inhibitors (6) and dual roles played by MMPs and TIMPs (7). The real conclusion came with the demonstration, against prevailing literature, that genetic deletion of MMP-9 or inhibition of MMP activity are not protective against experimental colitis in mouse models (8).

## What were the reasons to stop clinical studies of MMP-9 inhibition for IBD?

After the development of several different mouse monoclonal antibodies against MMP-9 (9, 10), the humanizaton of one mouse monoclonal antibody for clinical trials in cancer was started with the engineering of a chimeric monoclonal antibody (11), followed by safety and phase 2/3 studies in IBD (12). Similarly, with new techniques of immunization, additional monoclonal antibodies against MMPs were developed and tested in animal model studies (5). Although promising data were published, the clinical program of Andecaliximab (a chimeric recombinant IgG4 against MMP-9, formerly named GS-5745, Gilead Sciences) was stopped in IBD for futility reasons. In our own studies using mouse monoclonal antibodies against mouse MMP-9 (13, 14), we showed that parenteral injection of neutralizing antibodies against MMP-9 had clearly immunological effects that might become dangerous in clinical settings of endotoxinemia. Indeed, whereas MMP-9 gene-deficient mice showed protection against lethal endotoxinemia (15), the use of neutralizing mouse monoclonal antibodies against MMP-9 during endotoxinemia and during other situations with high serum levels of MMP-9 may result in detrimental effects. We hypothesize that, by the formation of MMP-9/anti-MMP-9 immune complexes, classical activation of complement system may enhance neutrophil and macrophage activation, resulting in more severe acute inflammation and further release of MMP-8 and MMP-9 and full activation of both pro-enzymes leading to further vascular leakage. In conclusion, our animal model studies did not provide evidence for a beneficial role of MMP-9 inhibition for IBD (8). Endotoxinemia, which may happen in IBD, remains a target for inhibition with synthetic MMP inhibitors (15), but may not constitute a good indication for MMP-9 inhibition with the use of neutralizing antibodies.

## An essential difference between exterior and interior barriers with consequences for MMP biology

The skin and mucosal membranes form a direct barrier between the outside world and the internal milieu. This implies that at the luminal side these organs are inhabited by micro-organisms, including bacteria and viruses, against which tight cellular and molecular barriers are needed to keep the basolateral side sterile. All internal organs are essentially sterile and, in addition, specialized organs, such as the brain and the eye have additional barriers to protect neurons against infections. These additional barriers are called, respectively, the blood-brain and the blood-retinal barrier and these consist of cellular (e.g. pericytes) and molecular (e.g. basement membrane) layers surrounding the vasculature.

We may compare and contrast the intestine, having a most extreme microbial content, that co-evolves with and helps the host with resorption of dietary products, with the eye that needs to remain aseptic at all instances and becomes completely or partially dysfunctional by the least infection. Such comparative studies help to think about alternative interpretations and to decipher how MMPs and TIMPs in normal and inflamed specialized tissues, such as eyes, may yield information about beneficial or detrimental functions to be considered in other maybe less complex organs.

**Figure 1:**
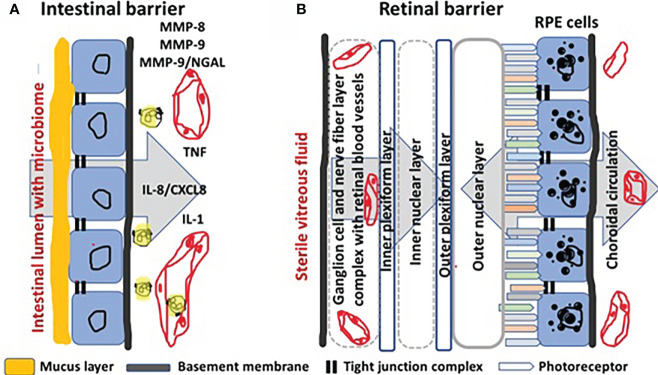
Comparison of intestinal with blood-retinal barrier. Panel **(A)**: The barrier of the intestinal epithelium protects the underlying tissue against invasion of microorganisms and diffusion of detrimental microbial molecules from within the lumen, while functioning mainly as area of transport of nutrients and fluid (large grey arrow on the background) into the subepithelial blood vessels (in red). The intestinal barrier is composed of tight junctions (indicated by two short black lines at the luminal side) between the epithelial cells (shown in blue) and by a basement membrane (indicated by the continuous subepithelial black line at the basolateral side). In addition, a mucus layer (in orange) produced by goblet cells (not shown) forms further protection against bacterial infection. In IBD, the pro-inflammatory cytokines tumor necrosis factor (TNF) and interleukin-1 (IL-1) induce locally the production of interleukin-8 (IL-8/CXCL8). IL-8 chemo-attracts neutrophils form capillaries and activates these cells to degranulate, yielding proteolysis by matrix metalloproteinases (MMPs), unencumbered by TIMP-1. Neutrophil collagenase/MMP-8 and gelatinase B/MMP-9 not only break down basement membrane collagens, but they also cleave the tight junction claudins and occludins. Neutrophils also produce calprotectin and neutrophil gelatinase B-associated lipocalin (NGAL) and covalent complexes with MMP-9 (MMP-9/NGAL). When the intestinal barrier is broken in the pathological conditions of IBD, neutrophil products may end up in the lumen and these may be detected in the faeces as IBD biomarkers. In Panel **(B)**, a comparison is made with the more complex barriers in the retina. The retina uses blood supply from two sources and, consequently, has two blood-retinal barriers (BRB). The inner retina derives blood from the retinal circulation (large grey arrow on the left side). The inner BRB is produced by the tight junctions between endothelial cells of the retinal circulation. The outer retina is supplied with blood from the choroidal circulation (bidirectional large grey arrow on the right side). The outer BRB is established by the tight junctions of the retinal pigment epithelial (RPE) cells (indicated as blue cells with black melanin granules). At the left side, the retinal blood vessels are separated from sterile vitreous fluid by the internal limiting membrane (continuous black line).

The comparison of barriers between intestine and retina at the histological and histopathological levels reveals similarities and differences, which may inspire further thinking about molecules and cells as possible therapeutic targets in IBD. In **Figure 1**, we outline some of these elements that are relevant in this comparison. In the intestine, the mucosal barrier consists of an epithelium with tight junctions and specialized goblet cells producing a thick layer of mucus helping to keep bacteria and other microorganisms, known as the intestinal microbiome, at the luminal side. How MMPs and TIMPs are expressed by specific cell types in healthy intestinal and IBD-injured mucosa (1, see Figure 4 therein), as well as critical MMP substrates that play roles in mucosal barrier destruction (reference 1, see Figure 3), were the subjects of illustrations in a previous review about IBD. Some essential molecular mechanisms and cellular contributors in the histopathology of injured IBD tissue are here schematically reiterated and complemented with insights from a review about cytokines in IBD (16) (**Figure 1A**). A normal intestinal microbiome is favorable for the host, because e.g. it helps with digestion of macromolecules, with biochemical conversions of essential nutrients and even with keeping pathogenic bacteria under control. The normal flux of nutrients in the intestine is from the luminal side through the mucus barrier and the epithelium towards the superficial blood vessels (**Figure 1A**, large grey arrow). However, when the integrity of the barrier is damaged, by either pathogenic microorganisms or by host factors, a cascade of events is triggered by exogenous endotoxins or lipopolysaccharides (LPS) or by endogenous tumor necrosis factor (TNF) and interleukin-1 (IL-1) being two master cytokine switches in IBD (16). TNF, IL-1 and also LPS induce locally and in many mononuclear cell types the production of the master chemokine IL-8/CXCL8, which is locally a major neutrophil chemoattractant and centrally a granulocytosis-promoting factor in humans (17) (*vide infra*). Activated neutrophils, e.g. by LPS or by IL-8/CXCL8, degranulate whereby sequentially and in an ordered way their contents are released. Within the present context, neutrophil collagenase/MMP-8 and gelatinase B/MMP-9 and neutrophil gelatinase B-associated lipocalin (NGAL) are produced by abundant neutrophils in IBD, in the absence of TIMP-1 (1). The MMPs enable neutrophils to degrade collagens in the extracellular matrix (ECM) and in basement membranes, as well as accessible tight junction proteins, that are also good substrates. This (i) leads to loss of the intestinal barrier; (ii) enables LPS to further diffuse to the basolateral side of the barrier, whereas (iii) neutrophil products, including calprotectin, NGAL, MMPs and NGAL/MMP-9 complexes may easily end up in the intestinal lumen.

In **Figure 1B**, a comparison is made with the structures forming the blood-retinal barrier (BRB). In the retina, two types of blood supplies co-exist and cooperate to provide this metabolically most active tissue with sufficient nutrients, while draining efficiently toxic compounds and cellular waste products, e.g. photochemical reactants and photoreceptor outer segments. The tight junctions between the endothelial cells of the retinal vessels constitute the inner blood-retinal barrier (iBRB), whereas the outer BRB (oBRB) is formed by the tight junctions between the retinal pigment epithelial cells. Diabetes induces retinal endothelial cell damage and breakdown of the iBRB, resulting in diabetic macular edema, ischemia and angiogenesis. Major functions of the retinal pigment epithelium (RPE) include oxygen, ion, nutrient and fluid transport to photoreceptors and outer and inner cell layers, phagocytosis of shed photoreceptor outer segments, retinoid conversion and storage and scattering of and protection against light (18). Although the structures and functions of the oBRB are well known, its possible roles in DR are less studied. In diabetes, hypoxia leads to neovascularization with proliferative diabetic retinopathy (PDR) as a common blinding complication (18). In the comparisons with the intestinal mucosa, it needs to be stressed that normal eyes are sterile and LPS-free. Another major difference is that in DR low grade inflammation is mediated mainly by mononuclear leukocytes, instead of neutrophils (19). Neutrophils produce masses of TIMP-free MMPs by degranulation in IBD, whereas in DR mononuclear leukocytes *de novo* synthesize limited amounts of MMP-9 in better balance with TIMP-1 (20).

Descriptions of MMP and TIMP expression in DR have been used to define whether these may act as biomarkers for disease (21, 22). This aspect is also valid for IBD (1) and additional literature is provided below. Aside MMP production levels, a second level relates to MMP functionality in DR. Indeed, in an animal model, it has been found that the neuroprotective molecule prominin-1/CD133 is a critical substrate of MMP-9 (23). This provides several reasons why inhibition of MMP-9, confined to the local and temporal environment of DR may be an ideal application of monoclonal antibodies neutralizing MMP-9 (24). In fact, inhibition of MMP-9 in DR with neutralizing antibodies may serve several functions. This may reduce neurodegeneration by leaving prominin-1/CD133 intact and it may result in restoration of the endothelial barrier in the retina. It is not excluded that local intraocular injection of neutralizing antibodies against MMP-9 may be helpful in diabetic macular edema and even replace anti-VEGF agents in those patients with diabetic macular edema, who do not respond to anti-VEGF therapy (25, 26).

With the extensive experience of using monoclonal antibodies against VEGF for the treatment of eye diseases, technical procedures for intravitreal injections to deliver monoclonal antibodies locally are well established and complications are rare. It is presently much more difficult to target MMP inhibitors to the intestine and certainly when inflammation is patchy. Recent developments to target gel-encapsulated drugs may come to the rescue (27). Adequate drug formulations may also include protease-activatable nanoparticles having targeting functions towards inflammatory sites. This strategy is also applied in cancer inflammation (28) thanks to the action of neutrophil-derived proteases. Cell-penetrating peptides may even deliver drugs from such nanoparticles to reprogram specific cell types (29). Indeed, the seeping of MMP-9/NGAL complexes, MMP-8 and MMP-9 into the lumen of the intestine may be used to localize such peroral drugs to the right sites. These aspects create already now opportunities to develop new systems for site-specific delivery of drugs. To obtain therapeutic benefit against local inflammation, the composition of compounds and timing of delivery are essential and this will be addressed further as “window of opportunity” below.

In conclusion, a simple environment for local drug delivery, such as the confined sterile vitreous fluid in the eye, constitutes already a challenge for the use of MMP inhibitors against inflammatory conditions in the acute phase. Therefore, diseases complicated by intrinsically infectious elements are more difficult to tackle, also because intestinal microbiota may modify intraluminal drugs (30).

## Biomarkers and MMP-based surrogate markers for IBD

Biomarkers are important to guide differential diagnosis, prognosis and therapy outcome. Although the investments in diagnostics research have always been inferior than those for therapeutics, new technologies of the “omics”-era bring diagnostics to a turning point, in which unbiased broad-spectrum approaches may yield alternative insights of sometimes previously wrong thinking and may contribute to the development of novel therapies. In IBD, an unmet need is the definition of predictive biomarkers for (non)response to therapy. In addition, the challenges in the biomarker field remain the same as at the time of single markers: cost, selectivity and accessibility.

Towards cost-efficiency, classical test systems such as commonly used ELISAs may be efficiently executed as two-in-one test. This is also a matter of complementation: two complementary tests make information content stronger. We reported on the detection of two biomarkers, MMP-9 and the neutrophil gelatinase B-associated lipocalin (NGAL) in IBD in one simple accessible ELISA and validated the data with the use of the labor-intensive and much more complex quantitative zymography test (31, 32). The value of two-in-one tests is still underestimated, because with a sandwich ELISA of the MMP-9/NGAL complex, these two markers may be specifically analyzed at once in clinical samples, including serum.

Regarding accessibility, stool and serum samples in IBD are easier to obtain than vitreous fluid of the eye in diabetic retinopathy. Faecal calprotectin is an established biomarker in IBD (33) and MMP-9 and lipocalin (NGAL) are being studied as surrogate markers for calprotectin (34). These molecules, as well as lactoferrin (35) and others are major products of neutrophils. For this reason, the presence of molecules in the faeces may be surrogate markers of neutrophils entering the intestinal lumen, where they disintegrate (*vide infra*). In recent clinical IBD studies, also serum biomarkers have been analyzed. The levels of cyclophilin A correlate with MMP-9 levels in UC, but not in Crohn’s disease (36). MMP-9 remains an excellent serum parameter for IBD disease activity (1, 31, 32, 37) and the reduction of TIMP-2 levels in serum predicts remission of IBD (38). In addition, neutrophil dynamics have been studied in UC (39) and these cells seem to remain key cells for biomarker production, including NOX1/NADPH oxidase production (40). Recently, with the use of ELISAs for MMP-9 and TIMP-1, protein levels were studied in sera from 35 children with UC and compared with those of a smaller control cohort. Serum levels were higher in patients with more extended and severe lesions and were correlated with C-reactive protein levels and medical scores, such as the Mayo score and the Paris Classification of the pediatric ulcerative colitis activity index (PUCAI) (41). In conclusion, the need for good biomarker research for IBD remains imminent and good combinations of fecal and serum tests, including the MMP-9/NGAL complex in patients and in animal models of IBD (31, 32, 42), may help in better stratifications to optimize IBD treatments.

## Insights into mechanisms of regulation of MMP-9 and therapeutic applications

MMP-9 interacts physiologically and pathologically with many other molecules in a network of proteases, intertwined with a network of inhibitors. MMP-9 has also many substrates and by its multidomain organization possesses many additional interactors (1, 43). We do not know the hierarchy of MMP-9 in this network. Therefore, it may have been wishful thinking that selectively blocking its activity with a monoclonal antibody would become a simple cure of IBD. The opportunities of today’s technologies, including single cell RNA sequencing and excellent immunophenotyping of all cell types from within a tissue, bring IBD research to the level that we may discover the molecular networks and hierarchies involved in IBD development, maintenance, progression and resolution. In the past, we have tried to upgrade scattered information about TIMPs and MMP-9 and other MMPs into logic frameworks (1, 43). The opportunities of these reviews have not yet been fully exploited. For instance, it is insufficiently recognized that the simple breakdown of gelatin by the gelatinases A (MMP-2) and B (MMP-9) only occurs AFTER collagens have been clipped by one of the collagenases (MMP-1, MMP-8 and MMP-13). For IBD research, with possibly a major role played by neutrophils, MMP-8 thus comes to the forefront. An opportunity for preclinical investigations thus is the testing of dual-specific inhibitors of both MMP-8 and MMP-9. Such reagents already exist (44) and these may form the basis for the development of new drugs.

In terms of regulation of MMP-9 levels and activities, during the last 5 years, a number of refinements have been added to the existing literature (1) about MMPs and TIMPs in IBD (**Table 1**). These data reinforce the opportunities for interferences at the regulatory and inhibitory levels. For the acute initiation or progression phases of IBD, the roles of p38 kinase and nuclear factor κB (NFκB) in breakdown of the intestinal barrier by substrate cleavage through MMP-9 (*vide supra*) have been reinforced (45, 47). In the chronic phase, when fibrosis is happening, the toll-like receptor (TLR) system with MyD88 as transducer towards the NFκB pathway seems not essential (47). In addition to regulation at mRNA level by genome-encoded small microRNAs (miRs) (reviewed in 43), long non-coding RNAs (lncRNAs) have recently been suggested to regulate MMP-2 and MMP-9 in pediatric IBD. Indeed, the growth arrest specific transcript 5 lncRNA was downregulated in inflamed *versus* normal biopsies, whereas those of MMP-2 and MMP-9 were increased (48). Finally, also with *in vitro* studies, progress at the regulatory level is made. With the use of monolayer cell cultures for the study of barrier integrity, it becomes possible to analyze complex mixtures with supposed beneficial effects (49, 50) and also to dissect thereafter which is the active compound or mixture of molecules involved.

**Table 1:** MMP regulatory studies, related to IBD since 2016.

Regulatory pathway	IBD study	Effect	reference
MyD88/NFκB	Intestinal fibrosis	Not essential for intestinal fibrosis	(47)
P38 kinase	Mouse gut permeability	Upregulation of MMP-9 contributing to permeability	(45)
lncRNA GAS5	Pediatric IBD	Negatively associated with MMP-9	(48)
Paeoniflorin	*In vitro* cell culture	Prevents intestinal barrier breakdown	(49)
NFκB and Notch	Traditional Chinese medicine	Leaves barrier proteins intact	(50)

In conclusion, regulation of MMP-9 in inflamed IBD tissue and in animal and cell culture models, seems to follow signaling pathways that were also described in cancer and other inflammatory diseases. The addition of lncRNA studies and the gradual move to define and study active compounds of traditional medicines and also food supplements and probiotics in well controlled ways deserve attention.

## Elements to consider for IBD treatments

**Figure 2:**
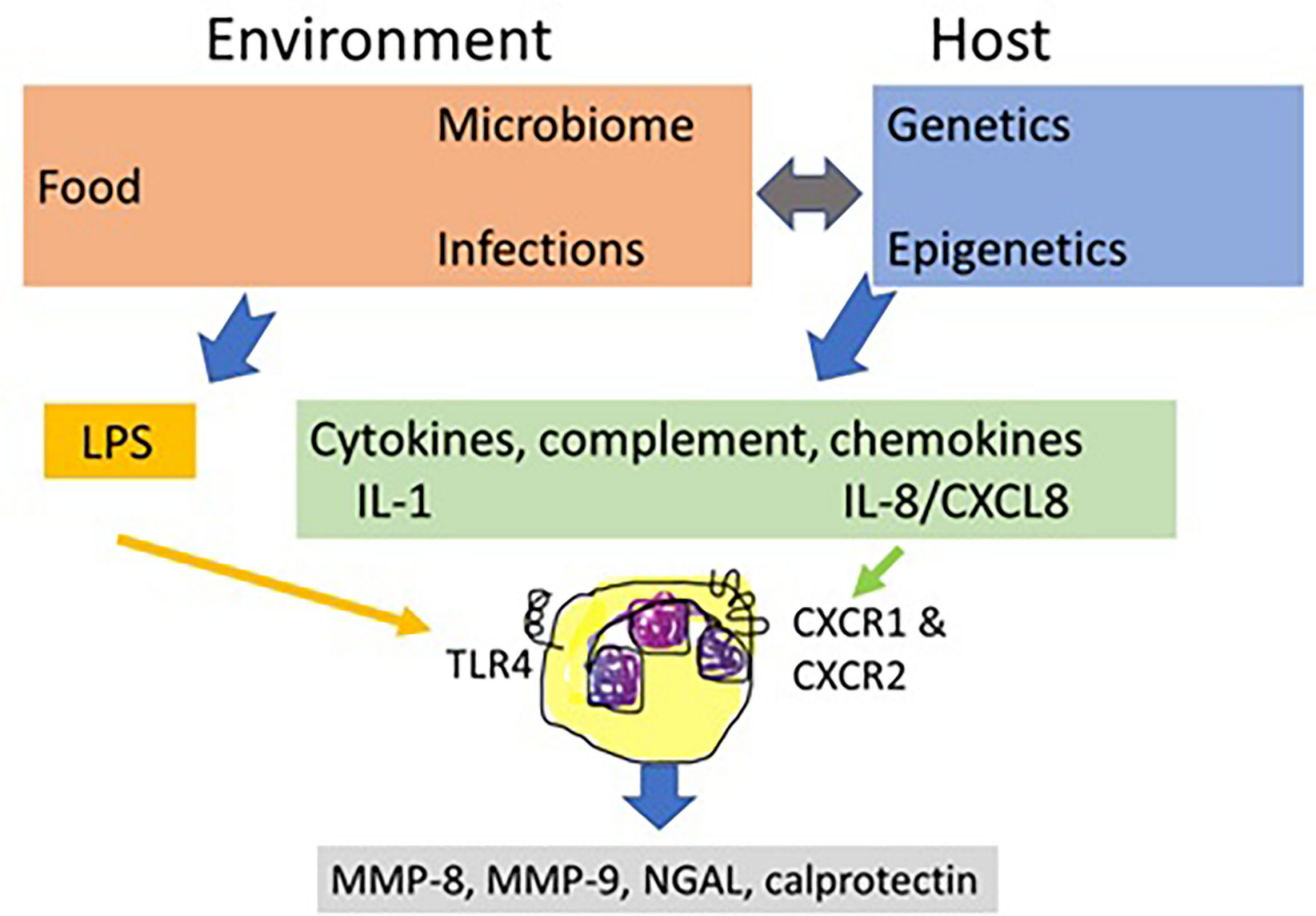
The central role of neutrophils in inflammatory bowel disease. Inflammatory bowel disease (IBD) biology is determined by host genetics and epigenetics and by environmental factors, including food, gut microorganisms and medication. Under balanced physiological conditions and under derailed conditions of infections and inflammations, these elements determine the control functions by the immune system. Neutrophils are critical cells in IBD biology. They respond to both environmental and host factors and play a central role by regulated release of MMPs and other granular contents. In IBD, the most prominent environmental factor is diet that influences the intestinal microbiome and, reciprocally, gut bacteria help with food processing and resorption. Dysregulations of the gut microbiome, occurring by infections, medication, food and by host factors, damage the intestinal barrier, enabling endotoxins or lipopolysaccharides (LPS) to seep across the damaged mucosal barrier. LPS directly activates local neutrophils through toll-like receptor 4 (TLR4) to degranulate and is also a key factor for indirectly modulating immune responses at various levels. IBD susceptibility, determined by host genetics and epigenetics (food, microbiota, medication, see double grey arrow) leads to inflammation with the production of immune mediators, including cytokines (e.g. IL-1 and TNF), complement factors (e.g. C3a and C5a) and chemokines, such as IL-8/CXCL8. Through their cognate receptors, for instance CXC chemokine receptor 1 (CXCR1) and CXCR2 for IL-8/CXCL8, these molecules also stimulate the release of neutrophil mediators. MMP-8 as a neutrophil collagenase clips and thereby denatures basement membrane and mucosal collagens. MMP-9 further digests denatured collagens and has many additional structural and functional substrates in the intestinal mucosa, including IL-8/CXCL8 and tight junction components. Aside MMP-8 and MMP-9, other neutrophil products, namely calprotectin and NGAL are additional biomarkers for the presence of neutrophil involvement in IBD, and these may all be detected in faeces samples.

The first aspect to consider is a more prominent role of neutrophils than hitherto recognized in the cell biology of IBD. In the circulation, these leukocytes outnumber considerably all other cell types, they play critical roles in all infections and inflammatory diseases and their numbers in the circulation are regulated by the primary cytokines TNF and IL-1, by chemokines such as IL-8/CXCL8 and by MMPs (51). In addition, neutrophils may be stimulated by many IBD-associated agonists, including viral- and bacterial-derived molecular patters acting through Toll-like receptors (TLRs) and formyl-peptide receptors, neurotansmitters and host molecules of the complement and chemokine systems. Host cytokines, such as IL-1 and chemokines are produced under genetic and epigenetic control, the latter being influenced by food, medication and normal and infectious microbiomes. The prototypic neutrophil agonist from abundant Gram-negative bacteria in the gut is lipopolysaccharide (LPS) or endotoxin and the prototypic host agonist is IL-8/CXCL8 (**Figure 2**). Collectively, these and many additional neutrophil agonists (e.g. complement C3a and C5a, bacterial formylpeptides) activate neutrophils to release MMP-8 and MMP-9 which affect the intestinal barrier and execute various feedback controls on cytokines, chemokines and their receptors. Neutrophils also secrete protective molecules including possibly calprotectin and NGAL. NGAL even occurs covalently associated with MMP-9 in the MMP-9/NGAL complex, which is still underestimated as an accessible biomarker for neutrophil involvement in IBD **(** **Figure 2**).

On the basis of insights into the molecular biology of MMPs and TIMPs, of the clinical spectrum of IBD phenotypes and various animal models of experimental colitis (1) and of the differences between acute (auto)-inflammatory and chronic autoimmune diseases, we may summarize that the clinical window of opportunity for IBD treatment by MMP inhibition is narrow in comparison with classical autoimmune diseases (52), and will, in particular, be confined to the neutrophil-driven acute inflammatory phase of IBD when MMP-mediated proteolysis is considerable and feedback inhibition by TIMP-1 almost non-existent. Aside future temporal and locoregional delivery of drugs affecting neutrophils and neutrophil-derived MMPs at the acute inflammatory sites in IBD, opportunities for MMP inhibition may also reside in chronic disease phases, e.g. in patients with fistulating disease or those developing fibrosis. In view of the finding that the hitherto best IBD biomarkers, including calprotectin, lipocalin and MMP-9/NGAL complexes are all neutrophil-derived, molecules that govern neutrophil chemotaxis to the intestine may also become therapeutic targets. In humans, IL-8/CXCL8 is the most potent neutrophil chemokine, acting through two receptors CXCR1 and CXCR2 **(** **Figure 2** **)**. It is relevant within IBD studies that MMP-9 potentiates IL-8/CXCL-8 at least 10-fold (53). The functional inhibition of MMP-9 will thus not only counteract intestinal barrier destruction, but also yield a functional reduction of local IL-8/CXCL-8-mediated neutrophil chemotaxis.

Unfortunately, long-term vision for the development of better animal models for some late and slow complications of IBD, i.e. fibrosis and fistulizing disease, is limited. Nevertheless, we feel that more and better preclinical research is needed before starting new clinical trials. Such approach is much better than placing patients at risk with possibly harmful treatments, based on insufficiently controlled gene knockout or inhibitor studies. In terms of neutrophil chemotaxis in mouse animal models of acute or chronic IBD, it is critical to know that IL-8/CXCL-8 does not exist in mice, and that the major neutrophil chemokine acting on both mouse CXCR1 and CXCR2 is granulocyte chemotactic protein-2 (GCP-2/CXCL6) (54, 55).

Another aspect to consider is the so called “window of opportunity” for the treatments of specific patients. This element, coming from rheumatology research and obviously useful for specific ophthalmological diseases (56), may be applied for IBD prevention and treatment, in particular for patients developing chronic complications of IBD or patients at risk who show to have increased faecal calprotectin levels or increased intestinal permeability.

## Therapeutic implications

During the last five years, our insights into the molecular biology of MMPs in IBD have been extended considerably with genetic knockout studies in mice, tests of small molecular weight inhibitors and highly specific monoclonal antibodies in animal model studies. Biomarker analysis is gradually moving to unbiased array-type techniques for the analysis of multiple analytes. The high information content of the latter will improve patient stratifications and be useful for the evaluation of new therapies. Here, we review recent developments about new therapeutics, address questions about therapy of chronic phases of IBD and illustrate the implications of parenteral use of antibody preparations in conditions with high protease loads, occurring in all types of inflammation.

Since 2016 (1), a limited number of new molecules have been tested in animal model studies of IBD (**Table 2**). These include betulinic acid (57), goat whey (58), polyphenols (59), eriocitrin (60), proglitazone (61), dietary products from grape seeds (62, 67) and lupin extracts (63) and traditional Chinese herbal preparations (64). In clinical studies, a cannabinoid receptor 2 agonist was found to improve mucosal healing (65). In line with the role of IL-8/CXCL8 in neutrophil chemotaxis, it is worthwhile to mention that an antagonist has beneficial effect in UC (66). Altogether this implies that with more and better controlled studies, clinical progress will continue for IBD. New treatments are needed, not only because prevention of IBD is not yet possible, but also because IBD is still a life-long debilitating disease, in particular for patients who become resistant to present therapies.

**Table 2:** Approaches for therapy delineated in (pre)clinical IBD studies, since 2016.

Compound	Animal model	Outcome	Ref.
Betulinic acid	Mouse DSS colitis	Decrease of colitis *in vivo*	(57)
Goat whey	Mouse DNBS colitis	Decrease of colitis *in vivo*	(58)
Polyphenols	Mouse colitis	Reduced colitis, no MMP-9 activity change	(59)
Eriocitrin	Mouse DSS colitis	Decrease of all severe clinical effects	(60)
Proglitazone	Mouse DSS colitis	Decrease of MMP-9 as colitis biomarker	(61)
Grape seed diet	Piglet DSS colitis	Barrier restauration with decreased MMP-9	(62)
Lupin extract	Mouse TNBS colitis	Less clinical signs and MMP-9 activity	(63)
Anti-oxydant	Chronic TNBS effect	Less clinical parameters and MMP-9 levels	(64)
	**Patient cohort**		
Cannabinoid RA	IBD	Increased mucosal healing	(65)
Il-8 antagonist	UC	Clinical improvement	(66)

Another pertinent question to resolve, before short- or long-term therapy with MMP-9 inhibitors is clinically tested, relates to whether MMP-9 is contributing mainly to acute inflammation in IBD or that it also contributes to chronic inflammatory and fibrosis processes and pathological angiogenesis, as are observed in diabetic retinopathy (24). A partial answer to this question was already provided above. In addition, a further complication in the immunobiology of MMPs and TIMPs is the observation that these molecules naturally exist as various proteoforms. In analogy with glycoforms, that are glycosylation variants of a single protein, proteoforms are the ensemble of all post-translationally modified versions and covalent complexes of a single protein. As an illustration, neutrophil-derived MMP-9 includes monomers and trimers and covalent complexes of pro-MMP-9 with NGAL (68). In addition, variants by glycosylation, nitrosylation and citrullination and proteolytically processed activation forms and degradation products may be isolated from *in vitro* cell culture supernatants and from tissue extracts and body fluids *ex vivo* (43). Individual proteoforms have different effects on blood vessels during the process of angiogenesis. For instance, the affinity between TIMP-1 and MMP-9 trimers is higher than that with MMP-9 monomers and TIMP-1 inhibits better *in vitro* angiogenesis by trimers than by monomers (68). Furthermore, MMP-9 trimers escape partially the inhibition by alpha2-macroglobulin, whereas monomeric MMP-9 is fully inhibited by alpha2-macroglobulin (69). Another so far neglected post-translational modification in IBD is citrullination (70). Recently, citrullination of MMPs was discovered (71) and demonstrated to be relevant in acute and chronic inflammations (71, 72). Whether posttranslational modifications of MMPs, including trimerization of MMP-9 and citrullination of various MMPs, have impacts on the processes of acute or chronic inflammation or fibrosis in IBD is not known, yet deserves attention. Imagine that specific MMP-9 proteoforms have beneficial effects and other ones possess detrimental functions, then the present duality of the studied mixtures may be dissected and monoclonal antibodies against specific MMP-9 proteoforms may become true drugs, also to improve IBD therapies. Another immunobiological consequence of the proteoform concept is the understanding that ELISAs for cytokines, chemokines and MMPs, which are commonly used for quantification in IBD clinical studies, always measure mixtures of the analytes, with varying affinities between the used antibody preparation and individual analyte proteoforms.

When using humanized and other monoclonal antibody-derivatives as therapy in milieus with considerable proteolysis, it is also relevant to know what the effect of proteolysis is on the immunoglobulin-derivatives, because this may influence responsiveness to therapy in individual patients. Indeed, Biancheri and colleagues showed that the anti-TNF preparations infliximab, adalimumab, and etanercept were cleaved by MMP-3 and MMP-12 and that by this action, etanercept lost its neutralizing activity (73). Surprisingly, it was demonstrated that MMP-9 does not cleave human IgG or IgM (74), which would constitute a benefit for the use of neutralizing antibodies against MMP-9 in conditions with considerable MMP-9 levels, such as IBD. Nevertheless and as explained above, MMP-9 inhibition in acute inflammation with major involvement of neutrophils is preferably done with dual-specific small molecular inhibitors that also inhibit MMP-8. The use of neutralizing antibodies leads to immune complex formation and possibly complement activation, which may aggravate inflammatory reactions. This effect may also be present and play a role in chronic diseases, including chronic stages of IBD and cancer. Along this line, it may be relevant to mention that recent oncology studies with Andecaliximab in cancer did not yield beneficial effects (75, 76).

## General conclusion

It is gradually recognized that the regulated expression of MMPs plays various roles in the pathogenesis, cycles of acute inflammation and resolution and chronic processes such as fibrosis and fistulating forms of IBD. The duality of MMP functions, beneficial *versus* detrimental, has not yet been sufficiently investigated, although this knowledge is well established since more than 5 years (1). Comparisons with what happens in other disease states, such as DR, arthritis and sepsis leads to the conclusion that MMPs have been insufficiently studied for their restorative role in IBD (77). From many association studies, it is clear that MMPs are excellent biomarkers, with the NGAL/MMP-9 complex as an exquisite example. By the concept of proteoforms, the functionalities of post-translational modifications of MMPs and the inherent consequence of the limitations of ELISAs as a quantitative test, a thorough revision of IBD immunology is necessary. However, these insights also provide the basis for better studies and create the hope to improve IBD therapies.

## Author contributions

GO wrote the first version of the review on the basis of literature searches and primary research performed in collaboration with both authors. SV shared promotorhips with GO in doctoral programs about inflammatory bowel diseases and contributed with critical additions to the review. AAE-A conducted research programs about diabetic retinopathy in collaboration with GO and added insights about inflammation in autoimmune diseases. All authors collaboratively wrote and approved the final version. They dedicate this work to the memory of Prof. Paul Rutgeerts, MD, PhD, FRCP, who pioneered clinical research activities at the University Hospitals of Leuven.

## Acknowledgments

The authors thank the members of their research teams for years of dedicated research. In particular the doctoral work by Magali de Bruyn, PhD, and help by Dr. Jennifer Vandooren, Dr. Estefania Ugarte-Berzal, Erik Martens and Pierre Fiten at the Rega Institute at KU Leuven, Belgium, are greatly appreciated. This study was supported by the Research Foundation of Flanders (FWO-Vlaanderen) and C1 Funding at KU Leuven and is dedicated to the memory of the late Professor Paul Rutgeerts, who started a school for IBD research at the University Hospitals in Leuven, Belgium.

## Conflict of interest

The authors declare that the research was conducted in the absence of any commercial or financial relationships that could be construed as a potential conflict of interest.

## Publisher’s note

All claims expressed in this article are solely those of the authors and do not necessarily represent those of their affiliated organizations, or those of the publisher, the editors and the reviewers. Any product that may be evaluated in this article, or claim that may be made by its manufacturer, is not guaranteed or endorsed by the publisher.
